# Mechanical and dentin bond strength properties of the nanosilver enriched glass ionomer cement

**DOI:** 10.4317/jced.55522

**Published:** 2019-03-01

**Authors:** Zahra Jowkar, Mohammad Jowkar, Fereshteh Shafiei

**Affiliations:** 1 Assistant professor, Oral and Dental Disease Research Center, Department of Operative Dentistry, School of Dentistry, Shiraz University of Medical Sciences, Shiraz, Iran; 2 Postgraduate Student, Department of Prosthodontics, School of Dentistry, Isfahan University of Medical Sciences, Isfahan, Iran; 3 Professor, Oral and Dental Disease Research Center, Department of Operative Dentistry, School of Dentistry, Shiraz University of Medical Sciences, Shiraz,Iran

## Abstract

**Background:**

The aim of this study was to investigate the mechanical properties and dentin microshear bond strength of a conventional glass ionomer cement (GIC) compared to GIC supplemented with silver nanoparticles (SNPs) at 0.1% and 0.2% (w/w).

**Material and Methods:**

SNPs were incorporated into a conventional GIC at 0.1% and 0.2% (w/w). The unmodified GIC was used as the control group. Compressive strength, flexural strength, and micro-shear bond strength (µSBS) to dentin were evaluated using a universal testing machine. Surface microhardness was determined using a Vickers microhardness tester. The data were analyzed using one-way analysis of variance (ANOVA) and Tukey’s test.

**Results:**

GICs containing 0.1% and 0.2% (w/w) SNPs significantly improved compressive strength, surface microhardness, and dentin µSBS compared to the unmodified GIC (*p*<0.05). A significant increase in the flexural strength was found for the GIC containing 0.2% (w/w) SNPs (*p*<0.05). However, the GIC containing 0.1% (w/w) SNPs did not affect flexural strength.

**Conclusions:**

GIC supplemented with SNP is a promising material for restoration because of its improved mechanical and bond strength properties. Therefore, it may be suggested for use especially in higher stress-bearing site restorations.

** Key words:**Glass ionomer cement, mechanical properties, micro-shear bond strength, silver nanoparticle.

## Introduction

Glass-ionomer cements (GICs) are widely accepted as dental restorative materials because of their unique properties such as chemical adhesion to dental tissues, fluoride releasing, low thermal expansion coefficient, and good biocompatibility (1). However, low wear-resistance, low fracture toughness, and high dissolution in water sorption are commonly considered as their most significant shortcomings which may lead to the growth of bacteria, secondary caries, and eventually restoration failure (1). In spite of fluoride release from GICs and their antibacterial effect, secondary caries have been reported as the main reason for GIC failure indicating that the antibacterial effect of GIC resulting from fluoride-release is not potent enough to inhibit bacterial growth (2). Bacteria may be present in the prepared tooth cavity after infected-caries removal based on the minimally invasive technique for tooth preparation. This technique may leave behind the caries-affected tissue in the cavity and thus increase the probability of the presence of residual bacteria at the prepared tooth cavity (3). Moreover, bacteria may invade the tooth-restoration interfaces during service if microleakage occurs in this area. Therefore, the longevity of the restoration might be jeopardized by secondary caries resulting from the colony growth of bacterial species, especially S. mutans, under the restoration (4). The primary and essential prerequisite for the development of cariopathogenic biofilms and caries is the adhesion of specific oral bacteria to tooth surfaces (5). Considering this fact, restorative materials should ideally have antibacterial activity to reduce the adhesion and proliferation of cariogenic bacteria at a very early stage and finally to decrease the occurrence of primary and secondary dental caries (5). Therefore, some efforts have been made to combat bacterial invasion and growth by incorporating antibacterial agents into restorative materials (1,6). In this regard, some filler particles have been incorporated into GICs to enhance their resistance to bacterial adhesion and solubility and also their mechanical properties (1,6). The filler incorporation into GICs should ideally improve the mechanical and antibacterial properties of GICs without interfering with their fluoride release and bond strength properties (1,7). It has been reported that the addition of chlorhexidine or its derivatives to GIC has improved its antimicrobial effect against cariogenic microorganisms. However, chlorhexidine incorporation has negative effects on the physical properties of GIC (8).

Recently, the use of nanoparticles (NPs) as fillers for restorative materials such as GICs has been explored mainly for the purpose of increasing their mechanical properties and antibacterial effects (1). Nanoparticles are insoluble particles with sizes smaller than 100 nm. The unique advantages of nanoparticles are their smaller-sized particles and the resultant higher surface area to volume ratio and also their stronger antibacterial activity than conventional fillers (9). Moreover, the probability that bacteria become resistant against metal nanoparticles is less than that in the majority of commercially available antibiotics (9). In this regard, different nanoparticles have previously been incorporated into dental materials (1,9). Hydroxyapatite and fluoroapatite nanobioceramics have previously been incorporated into conventional GIC which resulted in improved mechanical properties and bond strength to dentin (10). In addition, it has been reported that the incorporation of titanium dioxide nanoparticles into restorative GIC significantly improved antibacterial activity and physical properties such as flexural strength, compressive strength, and Vickers microhardness without compromising the bond strength of GIC to enamel and dentin (1). Also, neither hermetic growth stimulation nor cytotoxicity at lower concentrations of titanium dioxide nanoparticles was observed in culture with human gingival fibroblast (11). Additionally, GIC incorporated with titanium dioxide nanoparticles demonstrated acceptable to moderate biocompatibility in culture with human normal oral cells and human cancer cells (12).

Another nanoparticle which has been investigated in dentistry mainly because of its sustained ion release and the resultant long-term antibacterial property is silver nanoparticle (SNP). Moreover, 25-fold higher antibacterial efficacy than chlorhexidine has been reported for SNPs (13). SNPs have demonstrated broad-spectrum antibacterial and antiviral properties in low concentrations because of the multiple antibacterial mechanisms of silver such as adherence and penetration into the bacterial cell wall which result in increased cell wall permeability, the loss of the integrity of bacterial cell membrane, inactivation of the vital enzymes of bacteria, and loss of DNA replication ability (14). Moreover, SNPs have been found to be biocompatible especially in a lower concentration (15).

Although GICs containing antibiotics are recommended for the treatment of carious lesions to reduce the total number of viable bacteria, the addition of antibiotics should not jeopardize the biocompatibility or mechanical properties of GICs (1,7,16). It has been reported that the incorporation of SNPs into GIC at two different concentrations by weight (0.1% and 0.2%) does not affect the cytotoxicity of GIC (17). Additionally, enhanced antibacterial action has previously been reported with silver incorporation into a glass ionomer cement for cavity lining (16). However, to the authors’ knowledge, there is no published study investigating the effect of silver nanoparticle incorporation into GIC on the mechanical and bond strength properties of GIC. Therefore, the purpose of this study was to compare the mechanical properties (microhardness, flexural strength, and compressive strength) and the dentin bond strength of a conventional GIC to those of a conventional GIC modified with SNPs at 0.1% and 0.2% (w/w) concentrations.

## Material and Methods

A conventional GIC (GC Fuji II, GC Corporation, Tokyo, Japan) was used in this study. An SNP solution with the particle size of 20 nm (purchased from US-Nano materials Inc., USA) was added to the liquid of the GIC during the process to prepare specimens with two concentrations of silver by weight: 0.1% and 0.2% (w/w). The SNP solution was weighed carefully using a weighing machine with the accuracy of ±0.0001g (A&D, GR+360, Tokyo, Japan). The GIC specimens were divided into three groups for each test: GIC without SNPs (n=10); GIC with 0.1% SNPs (n=10); and GIC with 0.2% SNPs (n=10). The cements were prepared based on the manufacturer’s instructions. The preparations of the specimens for microhardness test, flexural strength, and compressive strength were based on the ISO 9917-1:2007 (18).

-Vickers microhardness test (VHN)

Thirty disc-shaped GIC specimens (9.5x1 mm) (n=10 for each group) with the recommended powder/liquid (P/L) ratio of 2.6/1 g were prepared following the manufacturer’s instructions in a Teflon mold according to ADA specification 27. The Vickers microhardness test was carried out using ISO 9001:2008 certified diamond indenter in a digital microhardness tester (SCTMC®, MHV 10002, China) with 10 N load and a dwell time of 10 s for 10 indentations across each specimen.

-Flexural strength

Thirty bar-shaped specimens (n=10 for each group) were prepared using a rectangular-shaped stainless-steel mold (25 mm length × 2 mm thickness ×2 mm width). The specimens were subjected to a three-point bending test in a universal testing machine (Instron, Z020. Zwick Roell, Germany) at a crosshead speed of 1 mm/min. Flexural strength, O´ (MPa), was calculated according to the following formula: O´=3Pl/2bd2 where P (N) is the load at fracture, l is the distance between the two supports (mm), b is the width of the specimen (mm), and d is the thickness (mm).

-Compressive strength

Thirty cylindrical specimens (n=10 for each group) prepared in a stainless steel mold (4 mm in diameter and 6 mm in height) were used to assess compressive strength. The compressive strength, CS (MPa), of the specimens was measured using the universal testing machine at a crosshead speed of 1 mm/min based on the following equation: CS=2P/πdh where P (N) is the load (N) at fracture and d and h are the diameter (mm) and thickness (mm) of the specimen, respectively.

-Microshear bond strength (µSBS) to dentin

**Figure 1:**
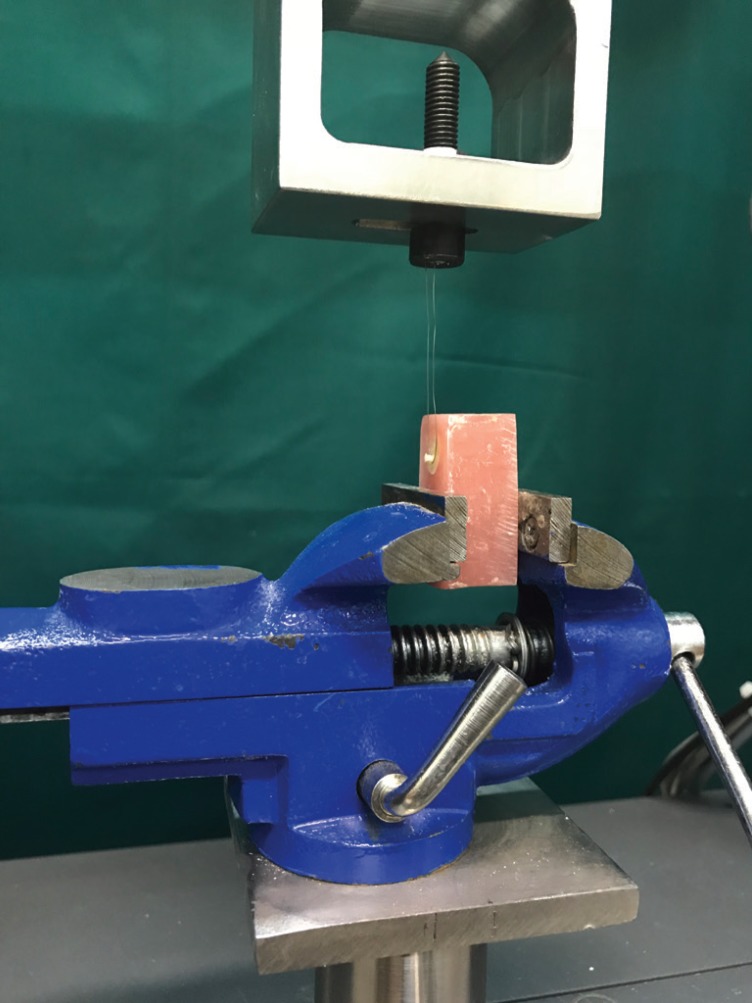
The wrapped stainless steel ligature around the base of the GIC micro-cylinder for measurement of µSBS.

Thirty caries-free extracted human third molars were collected and stored in 0.5% chloramine solution at 4°C for no longer than 1 month until use. The teeth were randomly divided into three groups (n=10). The occlusal enamel and superficial dentin were removed by sectioning the crowns using a water-cooled low-speed cutting machine (Mecatome T201 A, Presi, Grenoble, France) perpendicular to the long axis of the tooth and a flat mid-coronal dentin surface was exposed for each specimen. After fixing the specimens in acrylic resin (Acropars; Marlik Co., Tehran, Iran) with the dentin surfaces oriented perpendicular to the bottom of the mold, a uniform smear layer was created by slightly wet-grinding the dentin surface with 320-grit silicon carbide papers for 1 minute. Then, the specimens were rinsed and dried with an air-water syringe. The prepared dentin surfaces were treated with a cavity conditioner (Cavity Conditioner, GC Co, Japan) according to the manufacturer’s instructions. A piece of micro-tube from a micro-bore tygon tubing (R-3603, Norton Performance Plastic, Cleveland, OH) with the internal diameter of 0.7 mm and approximate height of 0.5 mm was placed on the bonding surface defined by an adhesive tape with a punched hole over the center of the flattened dentin surface and subsequently filled with GIC, GIC incorporated with 0.1% SNPs, and GIC incorporated with 0.2% SNPs (n=10 for each group). Immediately after bonding GIC to the dentin surfaces, the samples were stored in water at 37°C for 24 h. After removing the tygon tubing with a scalpel, µSBS to dentin was measured by a universal testing machine at the cross speed of 1 mm/min (MPa) (Fig. 1). The fracture load (N) was recorded and the µSBS (MPa) was calculated.

-Statistical analysis

Mean values and standard deviations for each test were measured. The obtained data were subjected to one-way analysis of variance (ANOVA) and multiple comparisons of Tukey’s test. All the analyses were performed using SPSS software version 17 (SPSS Inc, Chicago, USA) (*p*<0.05).

## Results

The means and standard deviations of Vickers microhardness (VHN), flexural strength (O´), compressive strength (CS), and micro-shear bond strength (µSBS) to dentin of the experimental groups are shown in  Table 1.

**Table 1:**
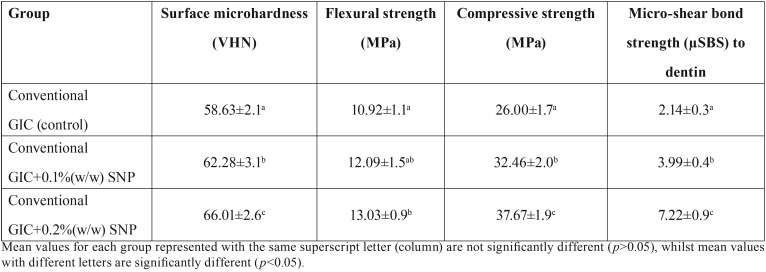
Means and standard deviations of Vickers microhardness (VHN), flexural (O´) and compressive strength (CS) and micro-shear bond strength (µSBS) to dentin of the experimental groups.

-Vickers microhardness

Surface microhardness (VHN) significantly increased with increasing concentrations of SNPs incorporated into GIC compared with the control group (*P* < 0.05). There was a significant increase in the surface microhardness for GIC+0.1 % (w/w) SNP and GIC+0.2 % (w/w) SNP groups (*P* < 0.05) compared to the control group. Also, the surface microhardness significantly increased for GIC+0.2 % (w/w) SNP group compared to GIC+0.1 % (w/w) SNP (*P* < 0.05).

-Flexural strength

The supplementation of 0.2% (w/w) SNP into GIC enhanced flexural strength (*p*<0.05) compared to the control group. There was no significant difference between the control and GIC+0.1 % (w/w) SNP groups (*P* > 0.05). Also, no significant difference was observed between GIC+0.1 % (w/w) SNP and GIC+0.2 % (w/w) SNP groups (*P* > 0.05).

-Compressive strength

The incorporation of 0.1% and 0.2 % (w/w) SNP into GIC significantly increased the compressive strength compared with the control group (*P* < 0.05). There was a significant increase in the compressive strength of GIC+0.2 % (w/w) SNP group compared to GIC+0.1 % (w/w) SNP group (*P* < 0.05).

-Micro-shear bond strength to dentin

Micro-shear bond strength to dentin increased significantly with increasing the concentrations of SNP into GIC compared with the control group (*P* < 0.05) ( Table 1).

## Discussion

The present study was carried out to investigate the effect of SNP incorporation into GIC on the mechanical and dentin bond strength properties. Two concentrations of SNPs (0.1 % and 0.2 % (w/w)) were selected for this study because a relatively low toxicity was reported after incorporation of these two concentrations of SNPs into GIC in a previous study (17). The best performance was shown for 0.2 % (w/w) SNP group in the current study.

Recently, minimal intervention dentistry has become more popular for caries removal during tooth preparation. However, this technique may increase the possibility of leaving more carious tissues in the tooth cavity containing active bacteria (3,19). Moreover, it is difficult to achieve a complete sealing of the tooth-restoration interface and micro gaps may form at the interface in the clinical practice (20). Therefore, restorative materials should ideally possess antibacterial properties to prevent bacteria-induced tooth sensitivity, pulpal irritation, and recurrent caries (5). An important point which should be considered when choosing an antimicrobial agent that may be added to restorative materials is that an ideal antimicrobial agent should provide effective antibacterial action without adversely affecting the mechanical and bond strength properties of restorative materials (1,6).

The application of nanoparticles represents an area of investigation that has recently attracted much attention in dentistry for controlling biofilm formation within the oral cavity because of the antibacterial, antiviral, anti-adhesive, and anti-inflammatory effects of nanoparticles (16). In this regard, metal-based nanoparticles such as SNPs have been used in various dental branches because of their broad-spectrum antibacterial properties (13,14). The mechanism underlying the antimicrobial effects of silver is still not fully understood. However, some possible explanations are as follows: (a) silver causes structural damage in bacteria by the production of reactive oxygen species including free radicals (b) the released biologically active silver ions can cause subsequent damage to DNA of bacteria and other phosphorus-containing compounds and inhibit their DNA’s ability to replicate (c) the direct contact of the very high concentration of silver particles with the cell wall can kill the cell. The main mechanism for the antibacterial activity of entrapped SNPs in resin materials is the last one (14,21-23). These properties have encouraged the use of SNPs in restorative dentistry (1,6,16). Two main strategies for using the antibacterial properties of nanoparticles such as SNPs in the oral cavity are combining dental materials with nanoparticles and coating the surfaces with nanoparticles to prevent microbial adhesion (24). Considering the inherent antibacterial properties of SNPs, the first mechanism was used in the current study and SNP was incorporated into a conventional GIC. The aim of this study was to investigate the role of SNPs incorporation in the mechanical properties and dentin microshear bond strength of GIC.

In spite of the fact that fluoride release from GICs renders them some antibacterial effects to suppress microleakage and secondary caries formation, it has been demonstrated that the addition of SNPs to GIC significantly improves its bactericidal activity against S. mutans (5,16). Moreover, a previous study assessed the cytotoxicity of conventional and resin-modified glass ionomer cements with and without the addition of SNPs. SNPs were added to these materials at two different concentrations by weight: 0.1% and 0.2%. It was found that SNP did not affect the cytotoxicity of the studied GICs (17). Therefore, two SNP concentrations (0.1 % w/w and 0.2 % w/w) were incorporated into a conventional GIC in the present study to investigate the effect of SNP incorporation on the mechanical and bond strength properties of GIC.

Compressive strength and flexural strength are considered as indicators for the load-bearing capacity of a restorative material in dentistry. Most of the masticatory forces are of a compressive nature. However, the exact critical value for a restorative material to be used in a stress-bearing area is unknown (1). In the present study, the GIC containing 0.1% and 0.2% (w/w) SNP exhibited a significantly higher compressive strength compared to the control group. Also, it was found that improvement in the flexural strength of GIC was significant at the concentration of 0.2% (w/w) SNP and 0.1 % (w/w) SNP did not negatively affect the flexural strength. These results may suggest the strengthening ability of SNP when added to GIC. The common reason for the low resistance of GIC to fracture is the presence of voids in the cement matrix which are formed by the inclusion of air during cement mixing. These voids may act as stress raisers and concentrators and eventually weaken the mechanical properties of the set cement (6). However, recent studies suggest that the voids tend to be filled with nanoparticles incorporated into GIC (6,25). The small sizes of the silver nanoparticles incorporated into GIC and the improved packing of particles within the matrix of the set cement may justify the improvement of the flexural and compressive strengths of the SNP-containing GIC. The incorporation of SNP into GIC may also result in a wider range of particle size distribution. Therefore, these small silver nanoparticles are able to occupy the empty spaces between the larger glass particles and may provide an additional bonding site for the polyacrylic polymer thereby reinforcing GIC (10,26). Moreover, the increased compressive strength may be attributed to the high density of interfaces of nanomaterial and the tendency of nanoparticles to resist the compression forces (27). The results of this study are similar to those of some previous studies. Incorporation of TiO2 nanoparticles into GICs improved compressive strength in a study by Elsaka *et al.* This finding was attributed to the small size of the nanoparticles and the effect of improved packing of particles within the matrix of the set cement (6). A higher compressive strength was also obtained following the incorporation of zirconium oxide and titanium dioxide nanoparticles into conventional GICs in a previous study (25). This result was explained by microscopic findings which showed the decreased occurrence of both air voids and micro-cracks within the set matrix of the GIC because of the improved homogeneity and increased consistency of GIC following the incorporation of the nanoparticles (25). This may be another explanation for the improvement of the mechanical properties of GIC after SNP incorporation in the present study.

A significant increase in the surface hardness of SNP-containing 0.1% and 0.2% (w/w) GIC compared to the control group was observed in the present study which may be a further evidence for matrix interaction. It seems that denser surface textures with fewer and smaller voids resulted in a higher hardness. A previous study has reported that the incorporation of 3% (w/w) TiO2 nanoparticles into GIC could improve the surface microhardness. A possible explanation for this increase in the surface microhardness is that fewer glass particles are present at the surface of GIC which results in a greater amount of acid to react with the nanoparticles. Moreover, the higher ratio of nanoparticles to the matrix at the interface resulting from the interstitial packing of the nanoparticles could have a role in the improvement of the microhardness (6).

Another finding in the current study is that the mean µSBS of the SNP-containing GIC to dentin significantly increased compared with the control group. GIC can chemically bond to tooth structure by the reaction of phosphate ions in the dental tissue with carboxylate groups in the polyacrylic acid. The type of dental substrate is among several factors that could influence the bond strength of glass ionomers to tooth structure (28). Dentin has a heterogeneous structure consisting of a complex inorganic/organic structure and consequently has a low surface energy (29). The results of the present study suggest that SNP addition not only does not interfere with the chemical bonding ability of GIC to dentin, but also may have a positive effect on the bond strength values.

Based on the results of the present study, SNP incorporation can improve the mechanical properties and bond strength to dentin of GIC. Moreover, visual inspection confirmed that the incorporation of 0.1 % (w/w) and 0.2 % (w/w) SNPs had no adverse effect on the color of GIC. However, the color of the samples was not assessed under a stereomicroscope. The results of the present study are in line with a previous study which has evaluated the effect of nanoparticle incorporation into GIC (1,6). The incorporation of titanium dioxide nanoparticles into a restorative GIC significantly improved antibacterial activity and mechanical properties such as flexural strength, compressive strength, and Vickers microhardness without compromising the adhesion to enamel and dentin in a previous study (1). Besides the positive effect of nanoparticles on the mechanical properties of GICs, a previous study showed that the incorporation of ZrO2 nanoparticles into GICs stimulated the adhesion of epithelial cells to the set cements (30). In the current study, the effect of SNP incorporation on the epithelial cell adhesion to GIC and the improved biocompatibility of SNP were not explored. These issues should be investigated in the future studies.

Based on the results of the present study, SNP-containing GIC is a promising restorative dental material especially for use in high-tension restoration considering the force of mastication. It has presented improved physical and bond strength properties. It can be suggested that this novel experimental GIC may be potentially useful for higher stress-bearing site restorations. However, further research on this material is warranted before its use in clinical practice. Further work is required to fully elucidate the effects of the incorporation of other types of nanoparticles into GIC in different concentrations and compare them with those of SNPs. Also, physicochemical surface interactions between the nanoparticles and the cements should be explored in the future. The present study has been carried out under in vitro conditions and there is a clear need for further in situ and in vivo studies. Moreover, the possible influence of nanoparticle incorporation on the setting time of GIC and the patterns of fluoride ion release from GIC should be investigated in the future. Also, the long-term bond strength, antibacterial and anti-caries properties of nanoparticle incorporation into GIC should be more elucidated in the future studies. The chemical interaction between SNPs and GIC composition should be assessed by specific analyses such as sophisticated spectroscopies and transmission electron microscopy (TEM) in the future.

Within the limitations of this study, we can conclude that SNP-containing 0.1% and 0.2% (w/w) GICs improved the compressive strength, surface microhardness, and µSBS to dentin compared to the unmodified GIC. The addition of 0.1 % (w/w) SNP to the conventional GIC did not compromise the flexural strength of GIC. The addition of 0.2 % (w/w) SNP to the conventional GIC improved the flexural strength of GIC.

## References

[1] Garcia-Contreras R, Scougall-Vilchis RJ, Contreras-Bulnes R, Sakagami H, Morales-Luckie RA, Nakajima H (2015). Mechanical, antibacterial and bond strength properties of nano-titanium-enriched glass ionomer cement. Journal of Applied Oral Science.

[2] Xie D, Weng Y, Guo X, Zhao J, Gregory RL, Zheng C (2011). Preparation and evaluation of a novel glass-ionomer cement with antibacterial functions. Dental Materials.

[3] Doozandeh M, Firouzmandi M, Mirmohammadi M (2015). The Simultaneous Effect of Extended Etching Time and Casein Phosphopeptide-Amorphous Calcium Phosphate containing Paste Application on Shear Bond Strength of Etch-and-rinse Adhesive to Caries-affected Dentin. The journal of contemporary dental practice.

[4] Kasraei S, Sami L, Hendi S, AliKhani M Y, Rezaei-Soufi L, Khamverdi Z (2014). Antibacterial properties of composite resins incorporating silver and zinc oxide nanoparticles on Streptococcus mutans and Lactobacillus. Restorative dentistry & endodontics.

[5] Bürgers R, Eidt A, Frankenberger R, Rosentritt M, Schweikl H, Handel G (2009). The anti-adherence activity and bactericidal effect of microparticulate silver additives in composite resin materials. Archives of Oral Biology.

[6] Elsaka SE, Hamouda IM, Swain MV (2011). Titanium dioxide nanoparticles addition to a conventional glass-ionomer restorative: influence on physical and antibacterial properties. Journal of dentistry.

[7] Dhull K, Nandlal B (2009). Comparative evaluation of fluoride release from PRG-composites and compomer on application of topical fluoride: An in-vitro study. Journal of Indian Society of Pedodontics and Preventive Dentistry.

[8] Palmer G, Jones F, Billington R, Pearson G (2004). Chlorhexidine release from an experimental glass ionomer cement. Biomaterials.

[9] Borzabadi-Farahani A, Borzabadi E, Lynch E (2014). Nanoparticles in orthodontics, a review of antimicrobial and anti-caries applications. Acta Odontologica Scandinavica.

[10] Moshaverinia A, Ansari S, Moshaverinia M, Roohpour N, Darr JA, Rehman I (2008). Effects of incorporation of hydroxyapatite and fluoroapatite nanobioceramics into conventional glass ionomer cements (GIC). Acta biomaterialia.

[11] Garcia-Contreras R, Scougall-Vilchis RJ, Contreras-Bulnes R, Kanda Y, Nakajima H, Sakagami H (2014). Induction of prostaglandin E2 production by TiO2 nanoparticles in human gingival fibroblast. In vivo.

[12] Garcia-Contreras R, Scougall-Vilchis RJ, Contreras-Bulnes R, Kanda Y, Nakajima H, Sakagami H (2014). Effects of TiO2 nano glass ionomer cements against normal and cancer oral cells. In vivo.

[13] Besinis A, De Peralta T, Handy RD (2014). The antibacterial effects of silver, titanium dioxide and silica dioxide nanoparticles compared to the dental disinfectant chlorhexidine on Streptococcus mutans using a suite of bioassays. Nanotoxicology.

[14] Rai M, Yadav A, Gade A (2009). Silver nanoparticles as a new generation of antimicrobials. Biotechnology advances.

[15] Gomes-Filho JE, Silva FO, Watanabe S, Cintra LTA, Tendoro KV, Dalto LG (2010). Tissue reaction to silver nanoparticles dispersion as an alternative irrigating solution. Journal of endodontics.

[16] Magalhães APR, Santos LB, Lopes LG, Estrela CRdA, Estrela C, Torres ÉM (2012). Nanosilver application in dental cements. ISRN Nanotechnology.

[17] Siqueira PC, Ana-Paula-Rodrigues Magalhães WC, Pires FCP, Silveira-Lacerda EP, Carrião MS, Bakuzis AF (2015). Cytotoxicity of glass ionomer cements containing silver nanoparticles. Journal of clinical and experimental dentistry.

[18] (2007). Dentistry-water-based cements-part 1: powder/liquid acid-base cements.

[19] Imazato S, Kuramoto A, Takahashi Y, Ebisu S, Peters MC (2006). In vitro antibacterial effects of the dentin primer of Clearfil Protect Bond. Dental Materials.

[20] Cheng L, Zhang K, Weir MD, Melo MAS, Zhou X, Xu HH (2015). Nanotechnology strategies for antibacterial and remineralizing composites and adhesives to tackle dental caries. Nanomedicine.

[21] Rai M, Deshmukh S, Ingle A, Gade A (2012). Silver nanoparticles: the powerful nanoweapon against multidrug-resistant bacteria. Journal of applied microbiology.

[22] Riad M, Harhash AY, Elhiny OA, Salem GA (2015). Evaluation of the shear bond strength of orthodontic adhesive system containing antimicrobial silver nano particles on bonding of metal brackets to enamel. Life Sci J.

[23] Yoshida K, Tanagawa M, Matsumoto S, Yamada T, Atsuta M (1999). Antibacterial activity of resin composites with silver-containing materials. European Journal of Oral Sciences.

[24] Hamouda IM (2012). Current perspectives of nanoparticles in medical and dental biomaterials. Journal of biomedical research.

[25] Gjorgievska E, Van Tendeloo G, Nicholson JW, Coleman NJ, Slipper IJ, Booth S (2015). The incorporation of nanoparticles into conventional glass-ionomer dental restorative cements. Microscopy and Microanalysis.

[26] Moshaverinia A, Ansari S, Movasaghi Z, Billington RW, Darr JA, Rehman IU (2008). Modification of conventional glass-ionomer cements with N-vinylpyrrolidone containing polyacids, nano-hydroxy and fluoroapatite to improve mechanical properties. Dental materials.

[27] Meyers MA, Mishra A, Benson DJ (2006). Mechanical properties of nanocrystalline materials. Progress in materials science.

[28] Magni E, Ferrari M, Hickel R, Ilie N (2010). Evaluation of the mechanical properties of dental adhesives and glass-ionomer cements. Clinical oral investigations.

[29] Lohbauer U (2009). Dental glass ionomer cements as permanent filling materials?–Properties, limitations and future trends. Materials.

[30] Semyari H, Sattari M, Atai M, Pournasir M (2012). The effect of nanozirconia mixed with glass-ionomer on proliferation of epithelial cells and adhesive molecules. Journal of Periodontology & Implant Dentistry.

